# Pharmacological Primary Cardiovascular Prevention and Subclinical Atherosclerosis in Men: Evidence from the Aragon Workers’ Health Study

**DOI:** 10.3390/jcm10050945

**Published:** 2021-03-01

**Authors:** Isabel Aguilar-Palacio, Sara Malo, Estibaliz Jarauta, Belén Moreno-Franco, Lina Maldonado, Luisa Compés, Mª José Rabanaque, José Antonio Casasnovas

**Affiliations:** 1 Preventive Medicine and Public Health Department, University of Zaragoza, 50009 Zaragoza, Spain; iaguilar@unizar.es (I.A.-P.); mbmoreno@unizar.es (B.M.-F.); rabanake@unizar.es (M.J.R.); 2 Instituto Aragonés de Ciencias de Salud, IIS Aragón, 50009 Zaragoza, Spain; estijarauta@gmail.com (E.J.); jacasas@unizar.es (J.A.C.); 3 Grupo de Investigación en Servicios Sanitarios de Aragón (GRISSA) IIS Aragón, 50009 Zaragoza, Spain; mlcompes@aragon.es; 4 Hospital Universitario Miguel Servet, IIS Aragón, 50009 Zaragoza, Spain; 5 Department of Economic Structure, Economic History and Public Economics, University of Zaragoza, 50009 Zaragoza, Spain; lmguaje@unizar.es; 6 Dirección General de Salud Pública, Gobierno de Aragón, 50009 Zaragoza, Spain

**Keywords:** subclinical atherosclerosis, primary prevention, pharmacological treatment

## Abstract

The objective of this study is to describe the profile of primary preventive treatment for cardiovascular disease in adult males and to analyze the association between treatment profile and subclinical atherosclerosis. We selected male workers who had undergone ultrasound imaging and had no previous history of cardiovascular disease (*n* = 2138). Data on the consumption of primary cardiovascular drugs from the previous year were obtained. We performed bivariate analyses to compare patient characteristics according to cardiovascular treatment and the presence of subclinical atherosclerosis, and logistic regression models to explore the association between these two variables. Among participants with no personal history of cardiovascular disease, subclinical atherosclerosis was present in 77.7% and 31.2% had received some form of preventive treatment. Of those who received no preventive treatment, 73.6% had subclinical atherosclerosis. Cardiovascular preventive treatment was associated only with CACS > 0 (odds ratio (OR), 1.37; 95% confidence interval (95% CI), 1.06–1.78). Statin treatment was associated with a greater risk of any type of subclinical atherosclerosis (OR, 1.73) and with CACS > 0 (OR, 1.72). Subclinical atherosclerosis existed in almost 75% of men who had no personal history of cardiovascular disease and had not received preventive treatment for cardiovascular disease.

## 1. Introduction

Cardiovascular disease is the main cause of morbidity and mortality in Spain and in developed countries [1]. Primary cardiovascular prevention strategies are based on the combination of a healthy lifestyle [2] and the use of preventive cardiovascular medication [3,4]. The use of hypotensive, lipid-lowering, antidiabetic, and antiplatelet drugs is effective in controlling cardiovascular risk factors and preventing cardiovascular disease [5]. 

However, atherosclerosis is typically a slow process that develops over several years before the appearance of clinical signs [6,7]. Few evidence-based studies have examined the correlation between subclinical atherosclerosis and subsequent development of cardiovascular disease. We recently described how subclinical atherosclerosis progresses relatively quickly in the short term [8]. It therefore seems plausible that cardiovascular medication could provide the greatest benefits if initiated during the early stages of subclinical arterial disease [9].

Prospective follow-up of the Aragon Workers’ Health Study (AWHS) [10] cohort for more than 10 years has yielded valuable information about the relationship between genetics, lifestyle, and cardiovascular disease risk factors. Follow-up of the cohort provides a window into the development and progression of cardiovascular disease from early subclinical stages to the onset of clinical manifestations such as cardiovascular health episodes. The information obtained from follow-up of the cohort is complemented with data on medication consumption, obtained from the pharmaceutical consumption database of the Aragon Health Department [11].

In the present study, we sought to characterize the profile of preventive treatment for cardiovascular disease in adult males with no history of clinical cardiovascular disease, and to evaluate the association between preventive treatment and subclinical atherosclerosis, as detected by vascular imaging.

## 2. Materials and Methods

### 2.1. Study Sample

We conducted a cross-sectional study within a worker´s cohort. Beginning in February 2009, workers from an automobile assembly plant in Zaragoza (Spain) were invited to participate in the AWHS, a cohort study that collected data gathered during the workers’ annual programmed health examination. After obtaining written informed consent, the research team collated clinical examination data, collected blood and urine samples, and provided participants with questionnaires on cardiovascular and lifestyle risk factors [10]. The study was approved by the Ethics Committee for Clinical Research of the regional government of Aragon [12].

We selected workers who had undergone an ultrasound examination between 2011 and 2014 and had no previous history of cardiovascular disease (*n* = 2259). Due to under-representation of the female sex in the cohort (*n* = 121), women were excluded from the analyses. The final sample consisted of 2138 men. Clinical data for the cohort are provided in Table 1. 

### 2.2. Subclinical Atherosclerosis Imaging

All AWHS participants aged 40–59 years were invited to undergo noninvasive imaging to detect subclinical atherosclerosis. Imaging consisted of ultrasound measurement of the carotid and femoral arteries and coronary computed tomography (CT) to calculate the coronary artery calcium score (CACS). The presence of plaques in both carotid and femoral arteries was determined using the Philips IU22 ultrasound system (Philips Healthcare, Bothell, Washington). Ultrasound images were acquired with linear high-frequency 2-dimensional probes (Philips Transducer L9-3, Philips Healthcare), using the Bioimage Study protocol for the carotid arteries [13] and a protocol designed specifically for the femoral arteries [14]. Inspection sweeps were obtained for the right and left sides of the carotid (common, internal, external, and bulb) and femoral territories. A plaque was defined as a focal structure protruding >0.5 mm into the lumen or reaching a thickness >50% of the surrounding intima or an intima-media thickness >1.5 mm. All measurements were determined using electrocardiogram (ECG)-gated frames corresponding to end-diastole (R-wave) [13]. 

CACS was obtained with a multidetector-row CT scanner (Mx 8000 IDT 16, Philips Medical Systems, Best, the Netherlands) using a low-dose, prospectively ECG-triggered, high-pitch spiral acquisition protocol. During a single breath hold, images were acquired from the tracheal bifurcation to below the base of the heart. Coronary calcium was quantified with calcium scoring software (Workspace CT Viewer, Philips Medical Systems) following the method of Agatston et al. [15], which calculates a summed score of all coronary calcified lesions, accounting for both the total area and the maximum density of coronary calcium.

The presence of plaque in the carotid-femoral territory and CACS were assessed in all subjects. CACS was represented as a categorical variable, classified into 3 groups (0, 1–99, and 100). Atherosclerosis was calculated as a summary measure, and was considered present in subjects with carotid or femoral plaques or a CACS > 0. 

### 2.3. Drug Consumption

Drug consumption data from the year prior to subclinical atherosclerosis imaging were obtained from the Aragon Medication Consumption Information System (Farmasalud) [11]. This system collects information on all outpatient prescriptions dispensed at pharmacies in Aragon through the public health care system. The following information is obtained from each dispensation: anonymous patient code; sex and birth date; anatomical therapeutic chemical (ATC) [16] code of the prescribed drug; dispensing date; number of defined daily dose (DDD); and number of packages dispensed. 

Data were collected for the following ATC codes (second level, therapeutic subgroup): antidiabetics (insulins and analogues (A10A), blood glucose lowering drugs excluding insulins (A10B) and other drugs used in diabetes (A10X)); antithrombotics (B01); antihypertensives (antiadrenergic agents (C02); diuretics (C03); beta blocking agents (C07); calcium channel blockers (C08); agents acting on the renin-angiotensin system (C09)); and lipid-lowering drugs (lipid modifying agents plain (C10A) and in combination (C10B)). For each of the four groups (antidiabetics, antithrombotics, antihypertensives, and lipid-lowering drugs), a participant was considered to be in treatment if they received at least 3 prescriptions for the same drug group within a 1-year period. 

Participants were divided into 3 groups according to their pharmacological treatment during the year preceding ultrasound evaluation: (1) No cardiovascular treatment: participants without 3 prescriptions for antidiabetics, antithrombotics, antihypertensives, or lipid-lowering drugs during the previous year; (2) Cardiovascular treatment: participants with at least 3 prescriptions for the same drug group during the previous year; (3) Statin treatment: participants with 3 prescriptions corresponding to ATC codes C10AA, C10BA, or C10BX during the previous year. 

### 2.4. Clinical Characteristics and Laboratory Data

Clinical and laboratory data were obtained from each worker’s annual medical examination. Clinical data included medical history, anthropometry, blood pressure, and heart rate. Blood pressure was calculated as the mean of 3 consecutive measurements obtained using an OMRON M10-IT automatic oscillometric sphygmomanometer (OMRON Healthcare Co. Ltd., Kyoto, Japan), with the participant resting in a seated position for 5 min between readings. Blood and urine samples were collected after overnight (>8 h) fasting and were processed for laboratory analyses the same day. Fasting serum glucose, triglycerides, total cholesterol, and high-density lipoprotein cholesterol (HDL-c) were measured by spectrophotometry (Chemical Analyzer ILAB 650, Instrumentation Laboratory SpA, Bedford, MA, USA). Levels of low-density lipoprotein cholesterol (LDL-c) were recorded. Whole blood glycosylated hemoglobin was measured by reverse-phase cation exchange chromatography and quantified by double wavelength colorimetry (ADAMS A1c HA-810 Analyzer, Arkray Factory, Minneapolis, MN, USA). 

Hypertension was defined as systolic blood pressure ≥140 mmHg, diastolic blood pressure ≥ 90 mmHg, or self-reported use of antihypertensive medication. Dyslipidemia was defined as total cholesterol ≥ 240 mg/dL, LDL-c ≥ 160 mg/dL, HDL-c < 40 mg/dL, or self-reported use of lipid-lowering drugs. Diabetes was defined as fasting plasma glucose ≥ 126 mg/dL or self-reported treatment with hypoglycemic medication [17]. Ever smokers were defined as individuals who currently or previously smoked. Former smokers were defined as individuals who smoked at least 50 cigarettes in their lifetime, but had not smoked in the last year. Obesity was defined as a body mass index (BMI) ≥ 30. Finally, cardiovascular disease risk was assessed for each subject using the European Systematic Coronary Risk Evaluation (SCORE) algorithm for low-risk cardiovascular disease countries [17,18].

All study procedures followed previously described standard operating procedures [10] and conformed to the ISO9001-2008 quality standard. 

### 2.5. Statistical Analyses

Descriptive analyses were conducted to describe the study population, the clinical characteristics of subclinical atherosclerosis, pharmacological treatment status, and subclinical atherosclerosis status. Data were expressed as mean and standard deviation (SD), or as percentages. The Kolmogorov–Smirnov test was used to asses normality. Bivariate analyses (U Mann–Whitney tests and chi square tests) were conducted to compare patient characteristics according to cardiovascular treatment (present/absent) and subclinical atherosclerosis (present/absent). Logistic regression models were performed to explore the association between cardiovascular preventive treatment (antidiabetics, antithrombotics, antihypertensives, or lipid-lowering drugs) and the presence of subclinical atherosclerosis. The association between statin treatment and subclinical atherosclerosis was also evaluated. Odds ratios (OR) and 95% confidence intervals (95% CI) were calculated for both crude and adjusted logistic regression models. 

All analyses were performed using R Studio Version 1.2.1335 (R Foundation for Statistical Computing, FreeSoftware Foundation, Boston, MA, USA).

## 3. Results

Our study population consisted of 2138 men with no previous history of cardiovascular disease. Table 2 shows the clinical characteristics of the study participants according to subclinical atherosclerosis status. CACS data were missing for 410 participants and atherosclerosis could be evaluated in 1985 participants. Of these men, 77.7% had atherosclerosis. Men with any form of atherosclerosis were older than those without (46.6 and 48.2 years, respectively) and had a higher frequency of cardiovascular risk factors. For all clinical characteristics studied, we observed significant differences (*p* < 0.05) between participants with atherosclerosis than those without, with the exception of obesity (*p* = 0.236). Within our study population, 10.7% of participants had a carotid plaque but not a femoral atheroma plaque, while 30.2% had a femoral but not a carotid atheroma plaque. The presence of cardiovascular risk factors increased in parallel with CACS values, with significant differences between CACS groups for all cardiovascular risk factors analyzed (*p* < 0.05).

Table 3 shows the clinical profile of the study participants according to pharmacological treatment. For all variables analyzed, there were significant differences between individuals who had received cardiovascular treatment and those who had not (*p* < 0.001). For all clinical conditions analyzed, we observed significant differences (*p* < 0.05) between individuals who had received statin treatment only and those who had not received any cardiovascular treatment. 

We examined the relationship between pharmacological treatment and subclinical atherosclerosis status. As shown in Table 4, 86.4% of participants who had received some type of cardiovascular treatment also had some form of atherosclerosis. Among those who received statin treatment only, this percentage increased to 90.0%. Comparison of participants who received cardiovascular treatment with those who did not revealed significant differences (*p* < 0.001) in the presence of any form of atherosclerosis and in CACS. This same comparison revealed no differences between groups in the presence of carotid atheroma only (*p* = 0.669) or femoral atheroma only (*p* = 0.793). Participants who received statin treatment only had a higher frequency of any form of atherosclerosis (*p* < 0.001) and higher CACS (*p* < 0.001).

Table 5 and Table 6 show the results of the multivariate analyses. Cardiovascular treatment was only associated with a CACS > 0 (*p* = 0.018) (Table 5). There was a significant association between smoking and all subclinical atherosclerosis-related parameters studied, with the exception of carotid atheroma only. When statin treatment was considered (Table 6), there was a positive association between statin treatment only and the presence of subclinical atherosclerosis (*p* = 0.022) and a CACS > 0 (0.018). 

## 4. Discussion

Of the participants in our study population with no personal history of cardiovascular disease, 77.7% had subclinical atherosclerosis. Those with subclinical atherosclerosis were older and had a higher prevalence of cardiovascular risk factors than those without. Among those with no personal history of cardiovascular disease, 31.2% had received some form of preventive treatment for cardiovascular disease during the year preceding the noninvasive imaging examination. Of those treated, 44.4% had been treated with statins. 

Subclinical atherosclerosis was present in 73.6% of workers who had received no preventive treatment for cardiovascular disease, and in 90% of participants who had been treated with statins. Multivariate analyses showed that treatment with any preventive cardiovascular treatment was associated only with a CACS > 0 (OR, 1.37; 95% CI, 1.06–1.78). Statin treatment was associated with a greater risk of any form of subclinical atherosclerosis (OR, 1.73; 95% CI, 1.10–2.83) and a CACS > 0 (OR, 1.72; 95% CI, 1.23–2.42). 

The prevalence of subclinical atherosclerosis in our study population was high. As described in previous studies [6], femoral atherosclerotic plaques are the atherosclerotic lesions most frequently detected by noninvasive diagnostic imaging, especially in ever smokers. We found that smoking was the most predictive factor associated with the presence of any atherosclerotic lesion in all arterial territories examined in our study. However, no such relationship was observed for carotid atherosclerosis; in fact, smoking appeared to protect against the development of this lesion. Nevertheless, this result may correspond to a spurious relationship since the group of subjects with carotid atherosclerosis showed the lower proportion of smokers in respect to the rest of groups. Further research is needed to determine whether additional factors not included in our model could have influenced these findings. Traditionally, high systolic blood pressure has been associated with thickening of the median layer of the arterial wall of the carotid territory as an adaptative response, and has a low predictive value for cardiovascular disease [19]. Hypertension has been associated with the progression and complication of atherosclerotic plaques prior to a clinical event, such as ischemic stroke [20]. This association could not be detected in our study, since the study population included younger individuals with less advanced lesions assessed by 2D ultrasound and the type of atherosclerotic lesion registered was the presence of plaque and no the increase of arterial intima-media thickness. Another factor of interest is lipoprotein (a). Lipoprotein (a) is a genetic marker that increases the risk of CVD due to its prothrombotic and proatherogenic properties. Its identification could be useful in stratifying cardiovascular risk in primary prevention, especially in young individuals [21].

Another factor associated with the presence of atherosclerosis, and in particular a CACS > 0, was previous treatment with statins. This finding is unsurprising, as treatment with statins has been associated with both the regression of non-calcified atherosclerotic lesions and the progression of CACS [22]. The most common lipid-lowering therapy, statin treatment, has been identified as the factor that most influenced cardiovascular disease prevention and regression of atherosclerosis [23]. However, this effect is achieved only by maintaining treatment with high intensity statins for at least 2 years [24]. Despite the high percentage of dyslipidemic participants in our study who were treated with statins, we observed considerable variability in the duration of treatment with lipid-lowering drugs, making it difficult to establish a causal link between statin treatment and atherosclerosis regression. Poor treatment adherence to statins has been widely described and is associated with poorer health outcomes and patient quality of life [25]. The use of new drugs, as PCSK9 inhibitors, have been presented as an opportunity to simplify pharmacological treatment and allow, therefore, an improvement of control and adherence to treatment. Nonetheless, these drugs are not currently being used in the AWHS cohort. Another aspect that must be considered is the low persistence to CV preventive treatments. In the case of lipid-lowering drugs, it has already been described in the AWHS cohort that less than 40% of patients were still persistent to treatment after one year of follow-up [26]. Additionally, the adequacy to CVD treatments is far from being optimal. Some previous analyses perform in this cohort show that, while the choice of statin treatment is associated with certain CVD risk factors, in a large proportion of cases it appears not to be based on the European clinical guidelines on CVD prevention criteria [27]. 

Noninvasive imaging revealed subclinical atherosclerosis in almost 3 out of 4 study participants who had no personal history of cardiovascular disease and had not received preventive treatment for cardiovascular disease. The low frequency of patients treated for cardiovascular disease risk factors is in line with previous reports [28,29]. In a previous study conducted in the AWHS cohort [30], we observed that many patients had not received any preventive treatment for cardiovascular disease, even when such treatment was warranted, and control goals were frequently not achieved. This lack of treatment has significant implications for the appearance of cardiovascular disease, and is striking given the context in which our study population was selected (i.e., favorable conditions for diagnosis and treatment including annual medical examinations and close contact between workers and medical staff). Based on these findings, it seems plausible that the proportion of individuals in the general population who have subclinical atherosclerosis and are not receiving any preventive cardiovascular treatment is even higher. Within the group of men with subclinical atherosclerosis, those who had not received preventive cardiovascular treatment were younger (47.7 years vs. 49.2) than those who had received treatment, and also had a markedly lower prevalence of cardiovascular risk factors. We observed significant differences (*p* < 0.001) between these 2 groups for the following parameters: HTA (22.4% vs. 72.0%), diabetes (2.3% vs. 14.4%), obesity (16.8% vs. 29.2%), and dyslipidemia (36.8% vs. 65.0%). Our findings show that even in individuals with a low prevalence of classical cardiovascular risk factors, there is a high risk of subclinical atherosclerosis, which is especially striking in the case of femoral plaques. Nonetheless, it should be noted that there are factors associated with the appearance of subclinical atherosclerosis that are not currently taken into account when deciding whether to prescribe this type of preventive treatment. In the view of the results obtained, the presence of subclinical atherosclerosis should be considered by clinicians to initiate pharmacological treatment as soon as possible, in order to mitigate their negative impact.

This study has some limitations that should be noted. Users of preventive cardiovascular treatments were classified based on data obtained from the Farmasalud database, which collects information on drugs dispensed through the public healthcare system. While Farmasalud does not collect information on prescriptions issued by private physicians or insurance companies, or in-hospital consumption, the data collected cover approximately 98.5% of the population of Aragon. Additionally, for the purposes of our study, we equated drug purchasing with drug consumption, which may not necessarily be the case, and may result in overestimation of the proportion of participants treated. Another issue that should be taken into consideration is the method used to define treatment. We classified a participant as treated if they had received at least 3 prescriptions for the same pharmacological group within a single year. Thus, a scenario could arise whereby a cardiovascular treatment is prescribed, but the patient does not take the drug, or does so sporadically. Further studies are required to determine why these men are not receiving preventive cardiovascular treatment, even when such treatment appears to be clinically indicated. The low number of women in the cohort made it impossible to perform a separate analysis of this group. Therefore, to avoid possible sex bias, this group was excluded from the analyses. Another important limitation is that CACS data were missing from 410 participants, which represents the 19.2% of the sample. These missing data also affected atherosclerosis calculation, as this is a summary measure. Nonetheless, even with a smaller sample, CACS showed a significant association with any type of CV treatment and with statin treatment. Finally, the fact that no noninvasive imaging examination was performed at the beginning of the study period meant it was not possible to establish whether subclinical atherosclerosis was already present when the participants began treatment. Additionally, the lack of imaging follow-up makes not possible to stablish the evolution of subclinical atherosclerosis at this moment, which is an important limitation. Currently, this cohort is undergoing a second imaging examination, which will allow us to analyze the evolution of subclinical atherosclerosis according to pharmacological treatment.

Strengths of the present study include the structure of AWHS cohort, which allowed us to analyze data from different sources (clinical and administrative), providing a broader perspective on primary prevention of cardiovascular disease. Furthermore, we used high quality data collection methods to obtain information on subclinical atherosclerosis. No differences have been reported between 2D and 3D ultrasound assessment of atherosclerosis [31]. 

## 5. Conclusions

In our study population, noninvasive imaging revealed subclinical atherosclerosis in almost three quarters of participants who had no personal history of cardiovascular disease and had not received preventive treatment for cardiovascular disease. Our findings underscore the need to revise and update classical cardiovascular risk factors in order to add new decision-making elements to cardiovascular disease management [5]. Subclinical atherosclerosis appears to be a useful parameter to include when evaluating personal cardiovascular risk in order to facilitate better treatment decisions. Finally, further research is required to establish the effect of cardiovascular preventive treatment on both the appearance and evolution of subclinical atherosclerosis. 

## Figures and Tables

**Table 1:** Clinical data for participants in the cross-sectional study.

Clinical Characteristics	N	%
Vascular imaging subcohort (men)	2138	100.0
Hypertension or hypotensive treatment	788	36.9
Diabetes or anti-diabetic treatment	116	5.4
Dyslipidemia or lipid-lowering treatment	905	42.3
Other CV prevention treatments (antithrombotic)	56	2.6
Any CV treatment (a, b, c, or d)	564	26.4
Obese (BMI ≥ 30)	429	20.1
Ever smokers	1583	74.0

N, number; CV, cardiovascular.

**Table 2:** Clinical characteristics of the study population.

Clinical Characteristics	No Atherosclerosis N = 442 (22.3%)	Any Form of Atherosclerosis N = 1543 (77.7%)	Carotid Atheroma Only N = 229 (10.7%)	Femoral Atheroma Only N = 645 (30.2%)	CACS		
					0 N = 1026 (59.4%)	1–99 N = 524 (30.3%)	>100 N = 178 (10.3%)
Age, mean (SD)	46.6 (4.2)	48.2 (3.7)	47.3 (4.1)	47.6 (3.9)	47.4 (4.0)	48.7 (3.3)	50.0 (2.3)
Ever smokers, N (%)	273 (63.2)	1220 (80.7)	150 (66.4)	509 (80.8)	736 (73.4)	415 (80.1)	150 (86.7)
Hypertension, N (%)	110 (25.3)	643 (42.5)	91 (40.3)	232 (36.8)	333 (33.2)	224 (43.3)	97 (55.1)
Diabetes, N (%)	12 (2.7)	102 (6.6)	7 (3.1)	40 (6.2)	41 (4.0)	32 (6.1)	24 (13.5)
Obesity, N (%)	81 (18.4)	321 (21.1)	50 (22.1)	135 (21.2)	197 (19.4)	103 (19.8)	48 (27.7)
Dyslipidemia, N (%)	136 (30.8)	720 (46.8)	95 (41.5)	279 (43.3)	394 (38.5)	237 (45.2)	98 (55.4)
SCORE value, mean (SD)	1.0 (0.7)	1.6 (1.2)	1.2 (0.8)	1.5 (1.3)	1.3 (1.0)	1.7 (1.1)	2.2 (1.7)
AWP, mean (SD)	95.2 (8.9)	97.1 (8.6)	97.2 (9.1)	97.2 (8.2)	96.3 (8.8)	97.1 (8.6)	98.1 (8.8)

CACS, coronary artery calcium score; SD, standard deviation; SCORE, cardiovascular risk based on the European Systematic Coronary Risk Evaluation algorithm; AWP, abdominal waist perimeter. Obesity is defined as a body mass index ≥ 30.

**Table 3:** Clinical characteristics according to pharmacological treatment status.

Clinical Characteristics	No CV Treatment N = 1472 (68.8%)	Any Type of CV Treatment N = 666 (31.2%)	Statin Treatment Only N = 296 (13.8%)
Age, mean (SD)	47.0 (4.2)	48.9 (3.4)	48.7(3.4)
Smokers, N (%)	1061 (73.4)	522 (80.8)	241 (83.4)
Hypertension, N (%)	314 (21.9)	474 (72.0)	151 (52.1)
Diabetes, N (%)	27 (1.8)	89 (13.4)	42 (14.2)
Obesity, N (%)	249 (17.1)	180 (27.7)	69 (23.9)
Dyslipidemia, N (%)	483 (32.8)	422 (63.7)	284 (95.9)
SCORE value, mean (SD)	1.2 (1.0)	1.8 (1.4)	1.8 (1.4)
AWP, mean (SD)	95.5 (8.5)	99.2 (8.7)	97.8 (8.3)

CV, cardiovascular; SCORE, cardiovascular risk based on the European Systematic Coronary Risk Evaluation algorithm; SD, standard deviation; AWP, abdominal waist perimeter. Obesity is defined as a body mass index ≥ 30.

**Table 4:** Pharmacological treatment and presence of subclinical atherosclerosis.

		No CV Treatment, Number (%)	Any Type of CV Treatment, Number (%)	Statin Treatment Only, Number (%)
No atherosclerosis		356 (26.4)	86 (13.6)	28 (10.0)
Any form of atherosclerosis		995 (73.6)	548 (86.4)	253 (90.0)
Carotid atheroma only		161 (10.9)	68 (10.2)	22 (7.4)
Femoral atheroma only		441 (30.0)	204 (30.6)	105 (35.5)
CACS:	0:	767 (64.5)	259 (48.1)	102 (43.8)
	1–99:	334 (28.1)	190 (35.3)	84 (36.1)
	>100:	89 (7.5)	89 (16.5)	47 (20.2)

CV, cardiovascular; CACS, coronary artery calcium score.

**Table 5:** Association between any type of cardiovascular treatment and the presence of subclinical atherosclerosis, adjusted by cardiovascular risk factors.

	Atherosclerosis		Carotid Atheroma		Femoral Atheroma		CACS > 0	
	Odds Ratio	*p*	Odds Ratio	*p*	Odds Ratio	*p*	Odds Ratio	*p*
Age	1.08 (1.05–1.12)	**<0.001**	0.99 (0.96–1.03)	0.701	0.99 (0.97–1.02)	0.662	1.11 (1.08–1.14)	**<0.001**
AWP	1.02 (1.00–1.04)	**0.029**	1.01 (0.99–1.03)	0.372	1.01 (1.00–1.03)	0.078	1.01 (0.99–1.02)	0.372
Hypertension (Ref. No hypertensive)	1.66 (1.25–2.22)	**<0.001**	1.17 (0.84–1.62)	0.362	0.90 (0.72–1.13)	0.384	1.25 (0.98–1.58)	0.068
Diabetes (Ref. No diabetic)	1.50 (0.80–3.07)	0.234	0.55 (0.22–1.14)	0.139	1.19 (0.78–1.81)	0.414	1.29 (0.83–2.02)	0.263
Obesity (Ref. No obesity)	0.68 (0.47–0.99)	**0.042**	0.96 (0.62–1.49)	0.870	0.90 (0.67–1.22)	0.515	0.86 (0.63–1.19)	0.377
Dyslipidemia (Ref. No dyslipidemia)	1.63 (1.28–2.10)	**<0.001**	0.94 (0.70–1.26)	0.685	1.01 (0.83–1.24)	0.896	1.17 (0.94–1.44)	0.160
Ever Smoker (Ref. No smokers)	2.35 (1.84–3.00)	**<0.001**	0.60 (0.45–0.82)	**<0.001**	1.52 (1.21–1.93)	**<0.001**	1.51 (1.18–1.94)	**0.001**
Any type of CV treatment (Ref. No CV treatment)	1.22 (0.89–1.70)	0.222	0.94 (0.65–1.36)	0.754	1.02 (0.80–1.31)	0.874	1.36 (1.06–1.76)	**0.018**

Ref., reference category; AWP, abdominal waist perimeter; CACS, coronary artery calcium score; Ref., reference category; CV: cardiovascular. All bold numbers means: statistically significant differences.

**Table 6:** Association between statin treatment only and the presence of subclinical atherosclerosis, adjusted by cardiovascular risk factors.

	Atherosclerosis		Carotid Atheroma		Femoral Atheroma		CACS > 0	
	Odds Ratio	*p*	Odds Ratio	*p*	Odds Ratio	*p*	Odds Ratio	*p*
Age	1.08 (1.05–1.12)	**<0.001**	0.99 (0.96–1.03)	0.675	1.00 (0.97–1.02)	0.691	1.11 (1.08–1.14)	**<0.001**
AWP	1.02 (1.00–1.04)	**0.022**	1.01 (0.99–1.03)	0.428	1.01 (1.00–1.03)	**0.058**	1.01 (0.99–1.02)	0.313
Hypertension (Ref. No hypertensive)	1.79 (1.33–2.43)	**<0.001**	1.08 (0.76–1.52)	0.653	0.96 (0.76–1.21)	0.752	1.33 (1.04–1.69)	**0.024**
Diabetes (Ref. No diabetic)	1.51 (0.80–3.10)	0.230	0.56 (0.23–1.16)	0.150	1.19 (0.77–1.80)	0.428	1.27 (0.82–2.00)	0.289
Obesity (Ref. No obesity)	0.68 (0.47–0.99)	**0.040**	0.97 (0.62–1.50)	0.879	0.90 (0.67–1.22)	0.509	0.86 (0.62–1.19)	0.373
Dyslipidemia (Ref. No dyslipidemia)	1.49 (1.15–1.93)	**0.003**	1.04 (0.76–1.42)	0.784	0.92 (0.74–1.15)	0.479	1.07 (0.85–1.35)	0.551
Ever Smoker (Ref. No smokers)	2.34 (1.83–3.00)	**<0.001**	0.61 (0.45–0.83)	**<0.001**	1.52 (1.20–1.92)	**<0.001**	1.50 (1.17–1.93)	**0.001**
Statin treatment only (Ref. No CV treatment)	1.74 (1.10–2.84)	**0.022**	0.68 (0.39–1.14)	0.155	1.29 (0.94–1.76)	**0.109**	1.72 (1.23–2.41)	**0.002**

Ref, reference category; AWP, abdominal waist perimeter; CACS, coronary artery calcium score; Ref., reference category; CV: cardiovascular. All bold numbers means: statistically significant differences.

## Data Availability

Data available under request.
